# Prospective cardiovascular magnetic resonance imaging in adults with Alström syndrome: silent progression of diffuse interstitial fibrosis

**DOI:** 10.1186/s13023-020-01426-4

**Published:** 2020-06-05

**Authors:** Shanat Baig, Rory Dowd, Nicola C. Edwards, James Hodson, Larissa Fabritz, Ravi Vijapurapu, Boyang Liu, Tarekegn Geberhiwot, Richard P. Steeds

**Affiliations:** 1 Department of Inherited Metabolic Disorders, Queen Elizabeth Hospital Birmingham, Birmingham, UK; 2 Institute of Cardiovascular Science, University of Birmingham, Birmingham, UK; 3 Department of Cardiology, Queen Elizabeth Hospital, Birmingham, UK; 4 Institute of Translational Medicine, Queen Elizabeth Hospital, Birmingham, UK; 5 Institute of Metabolism and Systems Research, University of Birmingham, Birmingham, UK; 6 Department of Cardiology, First Floor, Nuffield House, University Hospital Birmingham NHS Foundation Trust, Mindelsohn Way, Edgbaston, Birmingham, B15 2GW UK

**Keywords:** Alström syndrome, Cardiovascular magnetic resonance, Diffuse interstitial fibrosis, Progression, Extracellular volume, Ischaemic heart disease

## Abstract

**Background:**

Alström syndrome (ALMS) is a rare ciliopathy characterised by early onset insulin resistance, obesity, and dyslipidaemia and is a model for diseases that have huge social, health and economic impact. Cardiomyopathy develops in the majority, with high rates of morbidity and mortality, the definitive features of which are coarse replacement fibrosis and diffuse myocardial fibrosis (DIF). The pathogenesis of heart failure is thought to involve fibroblast accumulation and expansion of the extracellular matrix with excess protein deposition, leading to distorted organ architecture and impaired contractile function. Consecutive adults with genetically proven ALMS attending the National Centre for Rare Disease in Birmingham, England were studied. All patients underwent serial CMR, echocardiography and venous blood sampling, with computed tomography coronary angiography (CTCA) performed to assess severity of CAD. The aims of this study were: 1) to evaluate changes over time in DIF by cardiovascular magnetic resonance tissue characterization in ALMS; 2) to examine whether changes in DIF are associated with alteration in systolic or diastolic function; and 3) to evaluate the frequency and severity of coronary artery disease as a confounder for progression of ischaemic versus non-ischaemic fibrosis.

**Results:**

In total, 30/32 adults (63% male; 67% White British) participated. The median age at first scan was 21.3 years (interquartile range: 19.0–32.6) and participants were followed for a maximum of 67 months. Only 4 patients had significant coronary artery stenosis on post-mortem, invasive coronary angiography or CTCA. Mid short axis myocardial T1 times, myocardial extracellular volume, and left ventricular mass increased significantly over time, by an average of 21.8 ms (95% CI 17.4–26.1; *p* < 0.001), 1.1 percentage points (0.6–1.6, *p* < 0.001), and 2.8 g/m^2^ (1.9–3.7; *p* < 0.001) per year, respectively. These changes were not associated with significant deterioration in myocardial structure or function.

**Conclusions:**

This is the first comprehensive prospective study demonstrating progression of DIF in ALMS over time, although no structural or functional consequences were noted within a median three and a half years’ follow up. Further study is warranted to define whether DIF is a by-stander or the driver to impaired contractile function, heart failure and death.

## Introduction

Alström syndrome (ALMS) is a rare autosomal recessive cardiomyopathy (OMIM 203800) characterised by multiorgan disease. Infantile cardiomyopathy and retinal cone rod dystrophy are the earliest and most frequent manifestations of the syndrome. The most severe and often lethal complication of ALMS is heart failure due to cardiomyopathy [1]. Cardiomyopathy affects up to 65% of adolescents and adults, with high rates of morbidity and mortality [2]. Both the quality and length of life are reduced in adults with ALMS, and few survive beyond the age of 50 [2]. Autopsy data have demonstrated replacement myocardial fibrosis in non-coronary artery patterns, and diffuse interstitial fibrosis (DIF) has been detected on cardiovascular magnetic resonance (CMR) by elevation in T1 relaxation [3] and increased extracellular volume (ECV) in cross-sectional studies of ALMS [4]. These changes were associated with sub-clinical impairment of left ventricular (LV) function, assessed through change in myocardial strain. In many cardiovascular diseases, it has been postulated that the development of DIF over time may be a driver of abnormal myocardial deformation and diastolic dysfunction, leading ultimately to systolic heart failure [5]. While ALMS is rare, it offers a model of myocardial disease in which disease progression may be rapid, although serial assessment has not previously been performed.

Given the frequency of obesity, insulin resistance and metabolic syndrome in ALMS, ischaemic heart disease may well represent a potential confounder to increased T1 relaxation times and ECV [6]. Despite the high burden of CV risk factors, reported cases of coronary artery disease (CAD) are rare and data have been limited [7]. Moreover, defining serial changes in T1 and ECV relative to the frequency of coronary disease is important on a clinical basis, since primary and secondary treatment strategies differ. Therefore, the aims of this study were: 1) to evaluate changes in DIF over time by CMR tissue characterization in adults with ALMS; 2) to examine whether changes in DIF could be associated with alteration in systolic or diastolic function; and 3) to evaluate the frequency and severity of coronary artery disease as a confounder for progression of ischaemic versus non-ischaemic fibrosis.

## Methods

### Study design

Between March 2012 and January 2018, 32 consecutive adults with genetically proven ALMS attending the National Centre for Alstrom syndrome at the Queen Elizabeth Hospital Birmingham, United Kingdom (UK) were evaluated as part of standard clinical care. The clinic is unusual in that it is patient-centred, run in association with the charity Alström Syndrome UK and involves physicians from different specialties, nurses and relevant professions allied to medicine, forming a truly multidisciplinary team (MDT). Patients attend for 2 days and in this time, undergo all their relevant investigations, and are seen by all members of the MDT. At the first and subsequent visit, patients underwent annual disease monitoring following a standardized clinical protocol, including: biochemistry, echocardiography, and CMR. This was an observational, retrospective audit, utilizing existing data collected as part of clinical service. It was approved by local clinical governance committees (CARMS-15674) and conformed to the principles of Good Clinical Practice guidelines. Patients were eligible for inclusion in this audit if they were ≥ 16 years of age; had a genetically confirmed diagnosis of ALMS; and had been followed up in the service for a minimum of 1 year. Patients were excluded if they had an absolute contra-indication to CMR (1 patient with implantable cardio defibrillator) or a relative contra-indication for the study (1 patient was on a research drug trial). Otherwise, all adult patients in the service were included. Serial CMR and single computed tomography coronary angiography (CTCA) were included as standard for ALMS patients. Annual CMR was instituted from the start of the clinical service, while CTCA was introduced soon after the incidental finding of an occluded right coronary artery in an asymptomatic 38 year old male.

### Investigations

#### Blood markers

Venous blood samples were collected for haematology, biochemistry, and lipid profiles. Serum N-terminal pro B natriuretic peptide (NT-proBNP, ng/L) was measured by sandwich immunoassay with magnetic particle separation and chemiluminescent detection on an E170 analyser (Roche Diagnostics, Burgess Hill, United Kingdom) with a lower limit of detection of 0.6 pmol/L. Dyslipidaemia was defined as fasting cholesterol > 5 mmol/L and/or triglycerides > 1.7 mmol/L.

#### Echocardiography

Resting transthoracic echocardiography (TTE; ie33, Phillips) was performed by an accredited sonographer according to the British Society of Echocardiography minimum dataset [8]. Diastolic function was graded by an experienced cardiologist specialising in echocardiography (RPS) according to current guidelines [9].

#### Cardiac magnetic resonance imaging

Contrast enhanced CMR **(**1.5 T Avanto, Siemens Healthcare, Erlangen, Germany) was performed in-line with standard protocols to obtain LV and right ventricular (RV) dimensions, volumes, and mass on a bi-annual basis [10]. A steady-state free precession, single breath hold modified Look-Locker inversion recovery (MOLLI) sequence was used for T1 mapping in the basal and mid left ventricular short axis levels and the horizontal long-axis, before and 15–20 min after administration of gadolinium-based contrast agent (GBCA) [10]. Average scan parameters: 8 mm slice with a 192 read-out matrix, 6/8 phase partial Fourier with 81% phase resolution, field-of-view 320 × 320 mm^2^, repetition time 2.4 ms, echo time 1.01 ms, 11 phases [3, 3, 5 scheme], total breath hold 17 R-R intervals [11]. LGE imaging was performed 7–10 min after 0.15 mmol/kg of GBCA given as a bolus (Gadovist, Bayer Health Care) using phase sensitive inversion recovery. Analysis of volumes, mass, and function was performed off-line using Cvi42 software (Circle Cardiovascular Imaging, Calgary, Canada) according to the Society for CMR guidelines for reporting, with parameters indexed to height and body surface area (BSA), where appropriate [12]. Tissue tracking in the 3 long axis views was performed offline to assess 2D global longitudinal strain (GLS), according to previously published formulae [13]. Analysis of T1 was performed with manual contouring to define a region of interest (ROI) within the LV myocardium at basal and mid ventricular level (short-axis) on matched pre- and post-contrast images within the septum, as well as within the septum in the horizontal long axis view, and used to calculate myocardial extracellular volume (ECV) by validated formulae [14]. T1 values were measured in ‘normal myocardium’ defined by the absence of LGE. In addition, both short axis maps (basal and mid) were also manually contoured for endo- and epicardial borders, and partial voluming of blood minimised by using a 20% offset from the endo- and epicardial border [15]. Stability of T1 measurement over time was confirmed by weekly analysis of a phantom within the magnet [16]. Analysis of all CMR parameters was performed by a single observer (SB) blinded to the identity of the subject, date, and time. A randomly selected subset of 10 patients were selected for intra- and inter-observer reproducibility studies by SB and RV. Bland-Altman plots were used to assess the reproducibility of these measurements, with mean differences calculated to identify any bias. Agreement was further quantified using intraclass correlation coefficients (ICCs), using two-way random effects models with absolute agreement.

#### Phantom studies for T1 stability

T1 times stability over time was assessed using a phantom made of high density plastic beads of agar/nickle chloride in plastic tubes. High-density polyethylene macrobeads encased these plastic tubes arranged in a 3 × 3 array, with resin layer at the base. Each tube represented a range of T1 times. These were scanned regularly for 12 months on a fortnightly basis using the T1 protocol described above. A tight shim was set with all images acquired at iso-center. The analysis was done using ROI as described above.

#### Computed tomographic coronary angiography

Participants without renal dysfunction had a single CT coronary artery calcium (CAC) score and CTCA performed on dual energy source 64 multi-slice scanner (Siemens Somaton Definition, Erlangen, Germany) using retrospective electrocardiogram (ECG) gated acquisition. A 20 mL test bolus of contrast was used to set delay and 80 mL of contrast given for final image acquisitions. Significant CAD was defined as a lesion > 70% on post-mortem, invasive or CT coronary angiography.

### Statistical methods

The baseline demographics of the cohort were summarised, with continuous variables reported as means ± standard deviations (SDs) where normally distributed, and as medians and interquartile ranges (IQRs) otherwise. Changes over time in a range of markers were then assessed. Initially, Spearman’s correlation coefficients (rho) were produced to measure the correlation between each marker and the timing of the measurement, relative to each patient’s first scan. However, this approach did not account for the non-independence of the repeated scans on each patient. As a result, the analysis was repeated using a generalized estimating equation (GEE) approach. A separate model was produced for each marker, with the timing of the measurement, relative to each patient’s first scan as a continuous covariate, and the patient identification number (ID) as a nominal factor. The patient ID was set as the subject effect, with the within-subject effect being the timing of the scan, relative to the initial scan, rounded to the nearest 6 months. An autoregressive (AR) (1) correlation structure was assumed throughout. Analyses were then performed to assess whether changes over time in T1 corresponded to changes in other markers. For each marker, separate linear regression models were produced for each patient that had data with at least two scans, with the timing of the measurement relative to baseline, as a covariate. The gradients from the resulting models were then used to represent the rate of change in the markers and were compared using Spearman’s rho correlation coefficients. In addition, comparisons of gradients across dichotomous variables (e.g. gender) were performed using independent samples t-tests. All analyses were performed using IBM SPSS 22 (IBM Corp. Armonk, NY), with p < 0.05 deemed to be indicative of statistical significance throughout. For markers that were measured as percentages, gradients are reported as percentage point increases.

## Results

### Study population

There are 38 known adult ALMS patients within the United Kingdom, of whom 32 regularly attend the specialized service in Birmingham. Of these, two were excluded due to contra-indications to CMR (1 implantable cardioverter-defibrillator; 1 patient within research study), hence the analysis was based on the remaining 30. Baseline demographics and imaging data are presented in Table 1 and genetic data in Table 2. Family history of premature CAD was not recorded. The median age at the first scan was 21.3 years (IQR: 19.0–32.6), 63% of patients were male and 67% were of White British ethnicity. The first CMR scan was performed the day before or same day as the first clinical attendance in the service. From first CMR, the median follow-up time was 45 months (maximum 67 months), during which time 76 additional scans were performed, a median of 3 follow-up scans per patient, with 4 patients only having a single scan during the follow-up period. More than half the cohort had conventional cardiovascular risk factors (Table 1). At baseline, LV volumes, ejection fraction (EF) and mass were within normal limits for age, sex and body surface area on CMR, although GLS was low-normal and six patients (20%) had elevated NT-proBNP (≥144 ng/L). In total, 14 patients had evidence of LGE. Of these, 2 patients had LGE at the insertion of the RV into the basal inferoseptum (insertion point LGE), 1 had focal subendocardial (mid anteroseptal LV segment) and 2 had focal epicardial (1 basal and mid inferolateral; 1 mid inferior and mid inferolateral LV segments) LGE. Mid wall LGE was present in 5 patients located within the basal and mid inferolateral LV segments, while 4 patients had extensive LV and RV LGE. The LGE pattern did not correspond to a specific coronary artery disease distribution.

**Table 1:** Baseline Patient Demographic and Cardiovascular Imaging Data

**Factor**	**N**	**Statistic**
***Demographics***		
Age at first scan (years)	30	21 (19–33)
Male Gender (%)	30	19 (63%)
White British (%)	30	20 (67%)
BMI (kg/m^2^)	30	29 (26–32)
Systolic BP (mmHg)	30	130 ± 19
Diastolic BP (mmHg)	30	82 ± 12
Heart rate (beats/min)	30	86 ± 17
***CMR***		
T1 Basal (ms)	30	937 (913–1030)
T1 SAX Basal ECV (%)	30	27 (22–33)
LVEDVi (mL/m^2^)	30	58 ± 12
LVEF (%)	30	64 ± 9
LV mass index (g/m^2^)	30	55 (51–61)
LAV index (mL/m^2^)	30	34 (25–43)
GLS (%)	30	16 ± 3
***Echocardiogram***		
E/A ratio	29	1.5 (1.3–1.6)
Average E/e’ ratio	30	7.4 ± 2.1
e’ ave. (cm/s)	27	9.1 (7.7–11.0)
***Blood markers***		
Haemoglobin (g/dL)	30	14.0 ± 2.3
HbA1c (mmol/mol)	29	49 (39–71)
C-peptide (pmol/L)	30	3136 (1516–4113)
Cholesterol (mmol/L)	30	4.8 ± 1.3
Triglycerides (mmol/L)	30	2.5 (1.7–4.2)
NT-proBNP (ng/L)	30	51 (25–93)
eGFR (mL/min/1.73 m^2^)	30	108 (62–123)
***Comorbidities***		
Hypertension	30	17 (57%)
Dyslipidaemia	30	18 (60%)
Infantile cardiomyopathy	30	12 (40%)
Diabetes	30	18 (60%)
CKD	30	18 (60%)

*BMI* indicates body mass index, *BP* blood pressure, *CMR* cardiovascular magnetic resonance, *E/A ratio* mitral early filling (E)/atrial filling (A) ratio, *E/e’* mitral early filling (E)/early myocardial relaxation velocity (e’), *ECV* extracellular volume, *eGFR* estimated glomerular filtration rate, *GLS* global longitudinal strain, *HbA1c* glycated haemoglobin A1c, *IQR* interquartile range, *LAV* left atrial volume, *LV* left ventricular, *LVEDVi* left ventricular end-diastolic volume indexed to body surface area, *LVEF* left ventricular ejection fraction, *LVESVi* left ventricular end-systolic volume indexed, *NT-proBNP* serum Nterminal pro B natriuretic peptide, *RV* right ventricle, *SAX* short axis, *CKD* chronic kidney disease

Data are reported as N (%), mean ± SD, or median (IQR), as applicable

**Table 2:** Mutations in ALMS1 gene in study participants

**Mutation 1**	**Exome 1**	**Allele 1 nucleotide change**	**Allele_1 amino acid change**	**Homo/ Hetero/CH**	**Mutation 2**	**Exome 2**	**Allele 2 nucleotide change**	**Allele_2 amino acid change**
Nonsense	8	c.2041C > T	p.Arg681*X	Homo	?Nonsense	8	c.2041C > T	p.Arg681*
Nonsense	8	c.6823C > T	p.Arg2275*	CH	Nonsense	10	c.9535C > T	p.Arg3179*
Nonsense	8	c.2822 T > A	p.Leu941*	CH	Frameshift	16	c.10775delC	p.Thr3592Lysfs*6
Frameshift	8	c.6584delA	p.Lys2195Serf*10	CH	Nonsense	5	c.1008_1009delTG	p.Cys336fs*1
Nonsense	10	c.8002C > T	p.Arg2668*	CH	Nonsense	16	c.10879C > T	pArg3627*
Nonsense	10	c.9001C > T	p.Gln3001*	Hetero				
Frameshift	8	c.6895delG	p.val2299Trps*43	CH	Frameshift	16	c.11443C > T	p.Gln3815*
Nonsense	16	11107C > T	p.Arg3703*	Hetero				
Nonsense	16	11107C > T	p.Arg3703*	Homo	Nonsense	16	11107C > T	p.Arg3703*
Frameshift	16	c.10579_1580delAT	p.Met3527Valfs*13	CH	Frameshift	18	c.11856delC	p.Asn3952Lysfs*41
Frameshift	16	c.10769delC	p.Thr3590Lysfs*6	CH	Missense	8	c.5356A > G	p.Asn1786Asp
Nonsense	16	c.11107C > T	p.Arg3703*	Hetero				
Nonsense	8	c.6823C > T	p.Arg2275*	CH	Nonsense	10	c.9535C > T	p.Arg3179*
Frameshift	8	c.1729delA	p.Arg577Glyfs*17	CH	Nonsense	16	c.10477C > T	p.Gln3493*
	8	c6526C > T	p.Gln217*	Hetero				
Nonsense	10	c.8932C > T	p.Gln2978*	CH	Missense	8	c.5356A > G	p.Asn1786Asp
Nonsense	8	c.4937C > A	p.Ser1646*	Homo	Nonsense	8	c.4937C > A	p.Ser1646*
Nonsense	8	c.4937C > A	p.Ser1646*	Homo	Nonsense	8	c.4937C > A	p.Ser1646*
	9	c.7544-		Homo	Exon	9	c.7544-	
Deletion		200_7677 + 1110del			deletion		200_7677 + 1110del	
Nonsense	8	c.4937C > A	p.Ser2646*	Hetero	Nonsense	8	c.6526C > T	p.Gln2176*
Nonsense	8	c.6299C > A	p.Ser2100*	CH	Nonsense	16	c.10477C > T	p.GIn3493*
Nonsense	8	c.6299C > A	p.Ser2100*	CH	Nonsense	16	c.10477C > T	p.GIn3493*
Frameshift	16	c.10769delC	p.Thr3590Lysfs*6	CH	Missense	16	c.11410C > T	p.Arg38404*
Exon	9	c.7544-		Homo	Exon	9	c.7544-	
deletion		200_7677 + 1110del			deletion		200_7677 + 1110del	
Nonsense	8	c.2041C > T	p.Arg681*	Homo	Nonsense	8	c.2041C > T	p.Arg681*
Nonsense	8	c.2041C > T	p.Arg681*	Homo	?Nonsense	8	c.2041C > T	p.Arg681*
Exon deletion	9	c.7544-		Homo	Exon deletion	9	c.7544- 200_7677 + 1110del	
Nonsense	16	200_7677 + 1110del	p.Gln3495*	CH	Frameshift	16	c.10775delC	p.Thr3592Lysfs*6
Nonsense	16	11107C > T	p.Arg3703*	Hetero				
Frameshift	10	c.7911dupC	p.Asn2638Glnfs*24	Homo	Frameshift	10	c.7911dupC	p.Asn2638Glnfs*24

*CH* indicates compound heterozygote, Hetero, Heterozygote; Homo, homozygote

### Serial data

**Fig. 1:**
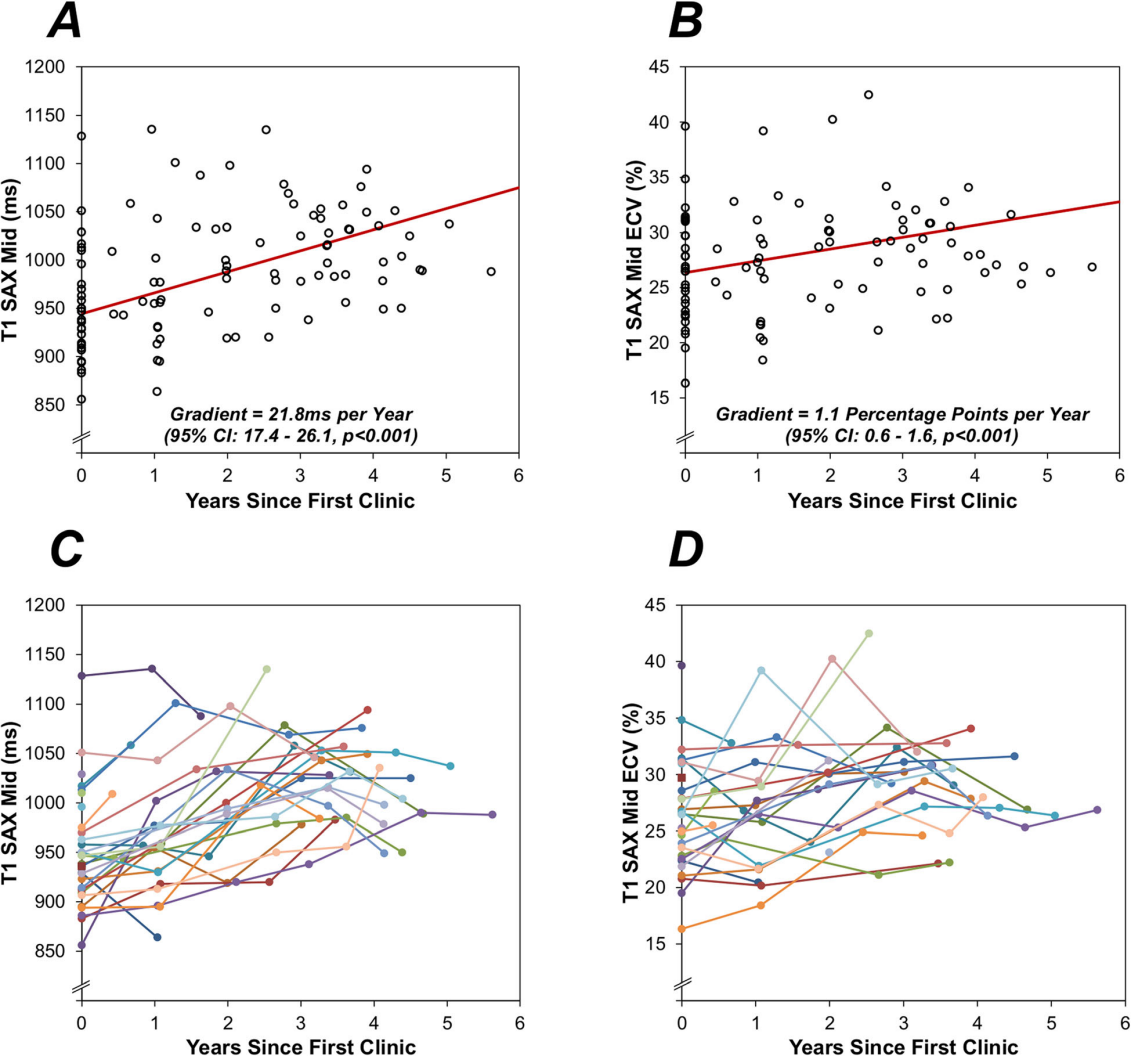
Serial Change Over Time

**Fig. 2:**
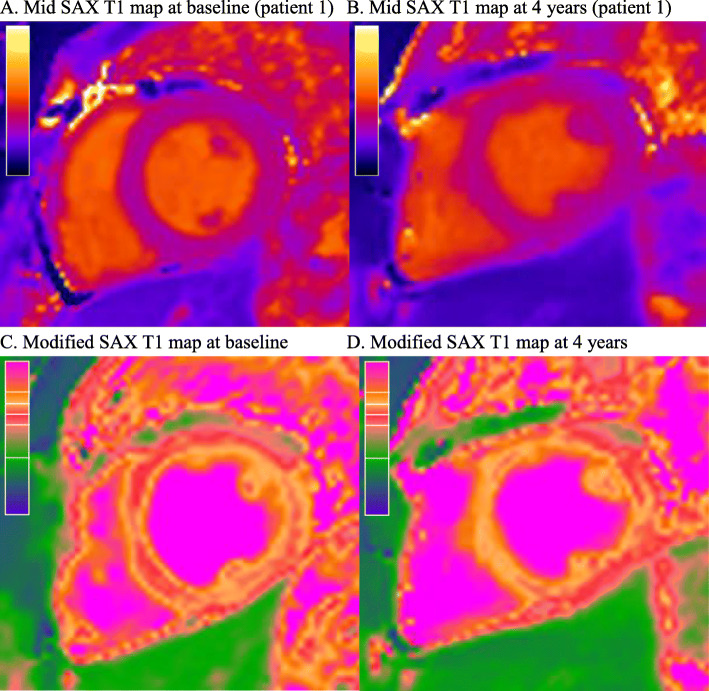
Changes in T1 Mapping Over Time Within an Individual. **a** Mid SAX T1 map at baseline (patient 1) **b** Mid SAX T1 map at 4 years (patient 1). **c** Modified SAX T1 map at baseline. **d** Modified SAX T1 map at 4 years. **a** represents T1 maps at baseline (T1 in ROI 923 ms) and **b** represent the increase T1 times at 4 years (T1 in ROI 1012 ms). The modified T1 maps (**c** & **d**) of the original maps (**a** & **b**) have been colour coded to show the difference. The scale represents an increase in T1 toward the top. SAX, short axis slice

On correlation analysis, T1 basal and mid ventricular level (short-axis; both p < 0.001), ECV basal and mid ventricular level (short-axis; *p* = 0.081 and p = 0.064), and LV mass (*p* = 0.008) all increased over time. Analysis of serial measurements using a GEE approach found significant increases over time in T1, as measured on the basal and mid ventricular (short-axis) levels, with similar gradients of 25.8 ms per year (95% CI: 20.0–31.7, *p* < 0.001) and 21.8 ms per year (95% CI: 17.4–26.1, *p* < 0.001), respectively (Fig. 1a). Significant increases in ECV were also observed, with gradients of 1.4 percentage points per year (95% CI: 0.8–2.0, *p* < 0.001) and 1.1 percentage points per year (95% CI: 0.6–1.6, *p* < 0.001) for basal and mid ventricular levels, respectively (Fig. 1b). Figure 2 shows an example of changes in T1 for a specific individual. In addition, the LV mass index was found to increase significantly over time, by an average of 2.8 g/m^2^ per year (95% CI: 1.9–3.7 g/m^2^, *p* < 0.001). Analyses using a correlation approach returned consistent findings (Table 3).

**Table 3:** Changes over time in markers

	***No. Patients***	***No. Scans***	**Correlation Analysis**		**GEE Analysis**	
			***Rho***	***p-Value***	***Gradient (95% CI)***	***p-Value***
T1 SAX Basal (ms)	30	104	0.415	**< 0.001**	25.8 (20.0, 31.7)	**< 0.001**
T1 SAX Mid (ms)	30	104	0.451	**< 0.001**	21.8 (17.4, 26.1)	**< 0.001**
T1 SAX Basal ECV (%, pp)	30	94	0.181	0.081	1.4 (0.8, 2)	**< 0.001**
T1 SAX Mid ECV (%, pp)	30	94	0.192	0.064	1.1 (0.6, 1.6)	**< 0.001**
LVEDV Index (ml/m^2^)	30	106	−0.113	0.247	0.04 (−0.60, 0.68)	0.909
LVESV Index (ml/m2)	30	106	−0.164	0.094	0.92 (−0.06, 1.90)	0.067
LVEF (%, pp)	30	106	0.104	0.289	−0.27 (−1.23, 0.68)	0.575
LV Mass Index (g/m^2^)	30	104	0.257	**0.008**	2.8 (1.9, 3.7)	**< 0.001**
LAV Biplane (ml/m^2^)	29	60	−0.221	0.090	−2.0 (−6.7, 2.7)	0.409
GLS	30	95	0.032	0.756	− 0.04 (− 0.33, 0.26)	0.809
GRS	28	86	0.010	0.924	−0.36 (−1.18, 0.47)	0.394
E/A	29	72	0.133	0.267	2.3 (−2.2, 6.9)	0.313
E/e’ Average	30	68	0.161	0.189	0.3 (−0.2, 0.8)	0.299
HBA1c (mmol/mol)	29	100	− 0.116	0.250	−0.9 (−2.5, 0.7)	0.284
C-Peptide (pmol/L)	30	94	−0.028	0.788	−90.7 (− 364.6, 183.2)	0.516
NTpro BNP (ng/L)	30	105	−0.101	0.306	34.4 (−33.9, 102.8)	0.324

Correlation analysis – results are from Spearman’s rho correlation coefficients between the marker and the timing of the scan, relative to the baseline scan

GEE analysis - results are from generalised estimating equation models, as described in the methods. The “gradient” represents the average yearly change in the marker over the period

Bold *p*-values are significant at *p* < 0.05. ECV and EF were measured as percentages, hence gradients represent percentage point (pp) increases

*E/A* mitral early filling (E)/atrial filling (A), E/e’, mitral early filling (E)/early myocardial relaxation velocity (e’), *ECV* extracellular volume, *HbA1c* glycated haemoglobin A1c, *LAV* left atrial volume, *LV* left ventricular, LVEDV index, left ventricular end-diastolic volume indexed to body surface area, *LVEF* left ventricular ejection fraction, *LVESV index*, left ventricular end-systolic volume indexed, *NT-proBNP* serum Nterminal pro B natriuretic peptide; SAX, short axis

Two patients developed LGE during follow up, 1 of whom developed new RV insertion point LGE and 1 developed subendocardial LGE in the mid anteroseptum (participants 10 & 11). No significant changes over time were detected in LV end-diastolic volume indexed to BSA (LVEDVi, *p* = 0.909), LV end-systolic volume indexed to BSA (LVESVi, *p* = 0.067), LV ejection fraction (EF, *p* = 0.575), left atrial volume indexed (LAVi, *p* = 0.409), or LV GLS (GLS, *p* = 0.809). In addition, no significant changes over time were noted in diastolic function on echocardiography, including mitral early filling (E)/atrial filling (A) ratio (p = 0.313), and average E/early myocardial relaxation velocity (e’, *p* = 0.299) (Table 3).

### Factors associated with change in ECV or T1

Changes in ECV and T1 were not found to be significantly associated with differences in LV volumes, mass, or EF over follow-up (Table 4). Although there was a significant association between increasing T1 and impaired (less negative) GLS (Rho 0.422, *p* = 0.036, this relationship was not mirrored by increasing ECV. Moreover, there was also no evidence of a significant association between the change in ECV or T1 and markers of diastolic function (E/A; E/e’ average; LAV). There was no evidence of a significant relationship between change in ECV or T1 with age, hypertension, diabetes, history of infantile cardiomyopathy, or presence/absence of coronary artery disease (CAD) (Table 5).

**Table 4:** Associations Between T1/ECV Over Time and Changes in Myocardial Structure and Function

**Gradient in**	**T1 SAX Mid Gradient**			**T1 SAX Mid ECV Gradient**		
	***N***	***Rho***	***p-Value***	***N***	***Rho***	***p-Value***
LVEDV Index (ml/m^2^)	26	0.019	0.927	24	−0.052	0.809
LVESV Index (ml/m^2^)	26	−0.444	**0.023**	24	−0.188	0.379
LVEF (%)	24	0.352	0.091	22	0.088	0.699
LV Mass Index (g/m^2^)	26	−0.056	0.784	24	0.203	0.340
LAV Biplane (ml/m^2^)	20	0.245	0.298	18	−0.377	0.123
GLS	25	0.422	**0.036**	23	0.024	0.914
GRS	23	0.268	0.217	21	0.116	0.618
E/A	22	−0.292	0.187	20	−0.029	0.905
E/e’ Average	23	−0.370	0.083	21	−0.268	0.241
HBA1c (mmol/mol)	24	−0.119	0.579	22	0.189	0.399
C-Peptide (pmol/L)	26	−0.101	0.624	24	−0.037	0.865
Pro NT BNP (ng/L)	26	−0.072	0.726	24	0.023	0.913

For each of the markers considered, a linear regression model was produced for each patient, with the timing of the scan, relative to the first scan, set as a continuous covariate. Only those patients with at least two valid scans for the stated marker were included in the analysis. Spearman’s (rho) correlation coefficients were then produced between the resulting gradients. Bold p-values are significant at *p* < 0.05

*E/A* mitral early filling (E)/atrial filling (A), E/e’, mitral early filling (E)/early myocardial relaxation velocity (e’), *ECV* extracellular volume, *HbA1c* glycated haemoglobin A1c, *LAV* left atrial volume, *LV* left ventricular, *LVEDV index* left ventricular end-diastolic volume indexed to body surface area, LVEF, left ventricular ejection fraction; LVESV index, left ventricular end-systolic volume indexed; NT-proBNP, serum Nterminal pro B natriuretic peptide; SAX, short axis

**Table 5:** Association between baseline factors and changes in T1/ECV

	**T1 SAX Mid Gradient (ms per Year)**			**T1 SAX Mid ECV Gradient (pp per Year)**		
	***N***	***Mean ± SD***	***p-Value***	***N***	***Mean ± SD***	***p-Value***
Age at First Scan*	26	−0.137*	0.505*	24	0.169	0.430
Ethnicity			0.278			0.360
*White British*	18	26.3 ± 25.2		17	1.8 ± 2.5	
*Other*	8	12.7 ± 35.9		7	0.7 ± 2.6	
Gender			0.112			0.864
*Male*	17	15.5 ± 29.4		15	1.4 ± 2.5	
*Female*	9	34.5 ± 24.8		9	1.6 ± 2.8	
ACE/ARB			0.969			0.771
*No*	6	21.7 ± 56.6		5	1.8 ± 2.9	
*Yes*	20	22.2 ± 16.0		19	1.4 ± 2.5	
Statins/Fibrates			0.103			0.121
*No*	14	30.7 ± 22.5		14	2.1 ± 2.8	
*Yes*	12	12.1 ± 33.1		10	0.5 ± 1.7	
Hypertension			0.930			0.117
*No*	11	21.5 ± 35.6		10	0.5 ± 2.4	
*Yes*	15	22.5 ± 24.2		14	2.1 ± 2.4	
Hyperlipidaemia			**0.040**			0.989
*No*	11	35.5 ± 21.8		11	1.5 ± 2.2	
*Yes*	15	12.3 ± 30.1		13	1.5 ± 2.8	
Infantile cardiomyopathy			0.710			0.904
*No*	16	20.4 ± 33.0		15	1.5 ± 2.9	
*Yes*	10	24.9 ± 22.0		9	1.4 ± 1.9	
Diabetes			0.764			0.132
*No*	9	19.7 ± 39.3		9	0.5 ± 2.5	
*Yes*	17	23.4 ± 23.0		15	2.1 ± 2.4	
Insulin resistance (not diabetes)			0.752			0.870
*No*	20	21.1 ± 30.2		18	1.5 ± 2.6	
*Yes*	6	25.5 ± 26.3		6	1.3 ± 2.4	
CKD			0.889			0.630
*No*	12	23.0 ± 33.3		12	1.2 ± 2.0	
*Yes*	14	21.4 ± 25.8		12	1.7 ± 3.0	
Presence of CAD**			0.622			0.121
*No*	20	23.5 ± 27.5		20	1.0 ± 2.0	
*Yes*	3	32.7 ± 44.7		3	3.4 ± 4.7	

Gradients were calculated on a per-patient basis, as described in the methods, with the resulting values reported as mean ± SD, and compared between groups using independent samples t-tests, unless stated otherwise. Only those patients with at least two valid scans for the stated marker were included in the analysis

*Reported as a Spearman’s rho correlation coefficient and p-value. **Excludes N = 3 patients with unknown CAD status. Note that ECV was measured as a percentage, hence gradients are reported in percentage points (pp) per year. Bold p-values are significant at *p* < 0.05

### Coronary artery disease

Presence or absence of CAD could not be established in 3 of the 30 patients. One patient was not assessed, as they refused to undergo CAC/CTCA due to a combination of claustrophobia and needle phobia, while 2 patients had not undergone CAD assessment at time of data collection. Of the remaining 27 patients, 3 patients presented with symptoms and were diagnosed with CAD. Of these, one died before a CTCA could be performed, but post-mortem showed a right coronary artery occluded with thrombus. The second patient had invasive coronary angiography following onset of symptoms and was found to have severe CAD. The third patient has CAD (> 70% stenosis in the mid left anterior descending artery) identified on CTCA and was treated by percutaneous coronary intervention with stent implantation. The remaining 24 asymptomatic patients all had a CTCA done, and 22 had CAC scoring. Of these, 4 were found to have elevated CAC Agatston scores of 48, 69, 156, and 209. These 4 patients were found to have mild atheroma with no flow-limiting stenosis, with CTCA being normal in the remainder. In summary, a total of 3/27 (11%) had significant CAD defined by > 70% stenosis, whilst a further 4/27 (15%) had non flow-limiting atheroma. Further details about these 7 patients are reported in Table 6.

**Table 6:** Details of Seven Patients with Coronary Artery Disease or Coronary Artery Atheroma

Patient ID	1	2	3	4	5	6	7
Age	38	42	19	48	36	43	19
Gender	M	M	M	M	M	F	M
Agatston	PM	ICA	1	48	69	156	209
Findings	RCA Occlusion	PCI to LAD	Mild atheroma	Mild atheroma	Mild atheroma	Mild atheroma	LAD stenosis
LGE Pattern	Extensive diffuse	Focal epicardial	Mid wall LGE	Extensive diffuse	Mid wall LGE	Extensive diffuse	Focal epicardial
LGE territory	Basal infero-lateral segment, basal, mid and apical lateral and inferior RV	Basal inferolateral and mid inferolateral walls.	Basal infero-lateral segment	Basal inferolateral and mid inferolateral transmural in the mid and apical anterior	Basal inferolateral	Basal and mid LV levels, mid antero-lateral, mid infero-lateral, mid inferior segments	Mid inferior and mid infero-lateral LV segments.
HTN	Y	Y	Y	Y	Y	Y	N
Hyperlipidaemia	Y	Y	Y	Y	Y	Y	Y
Diabetes	Y	Y	IR	Y	N	N	Y
CKD Stage	2	5	0	2	1	1	2
Infantile CM	N	Y	N	N	Y	Y	Y

*ICA* invasive coronary angiography, *CKD* chronic kidney disease stage, *CM* cardiomyopathy, *HTN* hypertension, *ID* identification number, *LAD* left anterior descending, *PM* Post mortem finding, *N* no, *Y* yes

### Intra- and inter-observer reproducibility, phantom study and Normal ranges

Ten CMR studies were randomly selected for assessment of intra- and inter-observer variation. There was no consistent pattern of inter-observer bias on Bland-Altman analyses. Intra-observer mean bias for basal T1 was 5.8 ms (95% CI -4.3, 15.8 ms), with an ICC (random model) of 0.9 (*p* < 0.001), whilst inter-observer mean bias for basal T1 was 6.4 ms (95% CI − 7.2, 20 ms), with an ICC of (0.9, *p* < 0.001). Normal T1 measurements and ECV values using the same protocol in normal volunteers on the same scanner were as follows: (n = 26) mid short axis (SAX) pre contrast T1 970 ± 11 ms; post contrast T1 522 ± 58 ms; ECV 25 ± 1%. Over 12 months, 25 MOLLI datasets were collected. The scanner room temperature was stable at 20.96 + 0.98 C. Coefficients of variation across the 9 tubes were stable and ranged from 0.436 to 0.872% when unadjusted for temperature and improved to and 0.436 to 0.868% with correction for room temperature.

## Discussion

This is the first prospective study to demonstrate an increase in T1 and ECV over time, paralleled by an increase in LV mass in ALMS. Despite changes in T1 and ECV, there was no association with serial changes in systolic or diastolic contractile dysfunction, either in terms of EF, E/A, E/e’, LA volume, or elevation in biomarker (NT-proBNP). Although increasing native T1 over time was associated with impaired (less negative) GLS, this was an isolated relationship that was not mirrored in relation to ECV and was not associated with other changes in functional parameters, The increased T1 and ECV were not significantly associated with the presence or absence of CAD. These data are consistent with mild, progressive myocardial fibrosis over time in ALMS, although no structural or consistent functional consequences were noted within a median three and a half years’ follow up. Why is this finding of importance? ALMS is characterized by obesity, insulin resistance and dyslipidaemia, diseases that are common but are projected to increase beyond that expected by evolving population demographics [17]. Furthermore, heart failure is highly prevalent in each, with adverse prognosis whether or not associated with coronary artery disease [18]. Given the premature onset of cardiomyopathy, ALMS is a paradigm to investigate the role of DIF in pathogenesis of heart failure, and whether this may be a target for therapeutic intervention [19] or simply a by-stander to other disease processes [20]. In such a population, particularly one with a high rate of hypertension at baseline, it is of interest that while indexed LV mass increased, there was no relationship with change in T1 or ECV. Our data identifies progression of DIF that is independent of coronary artery disease, but further research is needed to clarify relationship with functional decline.

Previous studies have demonstrated that T1 values and ECV are increased in ALMS compared to control subjects [3, 4]. These data are consistent with post-mortem histology studies demonstrating both coarse and DIF in adults with ALMS [1, 21, 22]. Although the data correlating high T1, increased ECV, and histology in ALMS are limited [4], T1 mapping and ECV on CMR as markers of DIF have been validated histologically in other conditions [23–25]. Longitudinal studies have shown T1 mapping to be a robust parameter, with little variability in healthy individuals over time, and in our study, the increase was measured in a single magnet using the same sequence with stable signal confirmed by phantom [26]. The increase in T1 and ECV was not, however, associated with a change in systolic or diastolic contractility, other than an isolated association between native T1 and GLS, measured either by CMR or echocardiography. In our previous cross-sectional study, an association was found between increased T1 and ECV and altered GLS measured using CMR tagging, and it may be that the method used in this serial study, tissue-tracking, was less sensitive [4]. It is also notable that in our previous and current work, a significant proportion of the ALMS population have both native T1 and ECV within normal range. In fact, baseline values in the current study for both native T1 and ECV were below the reference values for normal subjects, an effect which is usually attributable either to deposition of lipid or accumulation of iron, neither of which have been described in the limited histological data in adults with ALMS. Furthermore, the size of the change was small and it is possible either that change in function may require longer follow-up to have a significant impact, or that progression of fibrosis may be variable between individuals. This is consistent with lack of functional change on serial CMR over 2 years seen in patients with chronic stable cardiomyopathy who had a detectable increase in T1 times [27]. Given the potential signal of an association between increasing native T1 and impaired GLS, further prospective studies are needed to see if the increased T1 and ECV are associated in the longer term with the development of systolic dysfunction, heart failure and death, as has been demonstrated in other populations [28–31].

Infantile cardiomyopathy is a common finding in infants with ALMS, presenting within the first 3 months of life [32]. The majority (74%) survive, with response to standard heart failure therapy resulting in recovery of cardiac function in the majority [2]. Recent evidence suggests that the ALMS1 protein has a role in perinatal cardiomyocyte cell division and replication, and that its deficiency can cause mitogenic cardiomyopathy [33, 34]. The mechanisms leading to infantile cardiomyopathy appear to differ from those seen in adult cardiomyopathy, where myocardial fibrosis plays a dominant role. In keeping with this, there was no detectable difference in structure, function, or progression of T1/ECV between those patients with and those without a history of infantile disease. In addition, given the risk profile of the ALMS cohort under care, it was thought important to investigate the potential impact of CAD on the development of myocardial fibrosis and heart failure. More than a quarter of the cohort (7/27) had evidence of coronary atheroma, although only 3 patients had flow-limiting disease, 2 of whom were symptomatic. The prevalence of CAD was not found to influence the progression of T1 or ECV significantly in this study, although the statistical power of this analysis was low due to small numbers.

Limitations. The primary limitation of this study, inevitably, is the relatively small sample size. As a result of this, the statistical power of analyses is low, meaning that only relatively large effects would have been detecteable, increasing the risk of false negatives. This was especially applicable to the analyses comparing the T1 and ECV gradients across baseline factors, particularly for comparisons across groups with unequal numbers of patients, such as the presence of CAD (*N* = 3 vs. *N* = 20). Although numbers were small, participants had a genetically confirmed rare disease, recruited from a national centre with one of the largest adult cohorts available. Where significant trends over time were detected, the rates of change were generally small, and may not have been sufficient to have clinical impact in the short-term. However, the fact that the trajectories were similar across the whole cohort, and that testing of the scanner using a phantom within the magnet used demonstrated marked stability of the signal over time, would suggest that these trends are reflective of genuine progression of fibrosis. Further research is needed in a larger cohort, which is likely to require organisation of an international registry.

Baseline T1 values were heterogeneous in our cohort, consistent with our previous and other research in ALMS [3], although this may in part be accounted for differences in age, gender and co-existing metabolic status. This significant inter-individual variation suggests that development of fibrosis is not an inevitable consequence of the genetic defect alone and that environmental factors may also play an important role. We acknowledge however, the possibility of myocardial fat deposition or pseudo normalization of T1 due to the combined presence of lipid and fibrosis within the LV. Our own limited post-mortem data did not identify significant myocardial fat deposition in ALMS but recognise that further tissue characterisation, including T2 mapping, would have been enlightening. It is also important to note that distribution of mid wall LGE has a predilection for the inferolateral wall, which is seen in a number of different diseases, but is not limited to that location, as in early male Fabry disease, which is consistent with our hypothesis that fibrosis is not inevitable but may be subject to individual and environmental factors.

## Conclusion

This is the first comprehensive longitudinal study to suggest that myocardial fibrosis in ALMS progresses over time. There were no associated functional changes in systolic or diastolic function over follow-up, suggesting that these occur later in the natural history of ALMS cardiomyopathy. Longer term studies should be performed to confirm whether the development of DIF over time may be the primary driver rather than a consequence of ventricular dysfunction and heart failure.

## Data Availability

The datasets generated and/or analysed during the current study are not publicly available as the single centre location of the study, combined with the nature of the disease, mean that individual patients might be identified through the comprehensive data and material used in this study. These are are available from the corresponding author on reasonable request, subject to discussion on patient identifiable information.

## Abbreviations

A: Mitral atrial filling velocity
ALMS: Alström syndrome
AR: Autoregressive
BSA: Body surface area
CAC: Coronary artery calcium
CAD: Coronary artery disease
CMR: Cardiovascular magnetic resonance imaging
CTCA: Computed Tomography Coronary Angiography
DIF: Diffuse interstitial fibrosis
E: Mitral early filling velocity
e’: Early myocardial relaxation velocity
ECG: Electrocardiogram
ECV: Extracellular volume
EF: Ejection fraction
GBCA: Gadolinium-based contrast agent
GEE: Generalised estimating equation
GLS: Global longitudinal strain
ICC: Intraclass correlation coefficient
ID: Identification number
IQR: Interquartile range
LAV: Left atrial volume
LGE: Late gadolinium enhancement
LV: Left ventricle
EDV: End diastolic volume
EDVi: End diastolic volume indexed to body surface area
ESV: End systolic volume
ESVi: End systolic volume indexed to body surface area
MOLLI: Modified look-locker inversion recovery sequence
NT pro-BNP: N-terminal pro B natriuretic peptide
ROI: Region of interest
RV: Right ventricle
SAX: Short axis
SD: Standard deviation
TTE: Transthoracic echocardiography
